# An arbitrary waveform neurostimulator for preclinical studies: design and verification

**DOI:** 10.1007/s11517-024-03241-6

**Published:** 2024-12-12

**Authors:** Hipolito Guzman-Miranda, Alejandro Barriga-Rivera

**Affiliations:** 1 Department of Electronic Engineering, Universidad de Sevilla, Camino de los Descubrimientos, S/N, 41092 Sevilla, Spain; 2 Department of Applied Physics III, Universidad de Sevilla, Camino de los Descubrimientos, S/N, 41092 Sevilla, Spain; 3 School of Biomedical Engineering, University of Sydney, Camperdown, NSW 2050 Australia

**Keywords:** Neural stimulation, Retinal prosthesis, Arbitrary current waveform, FPGA

## Abstract

Neural electrostimulation has enabled different therapies to treat a number of health problems. For example, the cochlear implant allows for recovering the hearing function and deep brain electrostimulation has been proved to reduce tremor in Parkinson’s disease. Other approaches such as retinal prostheses are progressing rapidly, as researchers continue to investigate new strategies to activate targeted neurons more precisely. The use of arbitrary current waveform electrosimulation is a promising technique that allows exploiting the differences that exist among different neural types to enable preferential activation. This work presents a two-channel arbitrary waveform neurostimulator designed for visual prosthetics research. A field programmable gate array (FPGA) was employed to control and generate voltage waveforms via digital-to-analog converters. Voltage waveforms were then electrically isolated and converted to current waveforms using a modified Howland amplifier. Shorting of the electrodes was provided using multiplexers. The FPGA gateware was verified to a high level of confidence using a transaction-level modeled testbench, achieving a line coverage of 91.4%. The complete system was tested in saline using silver electrodes with diameters from 200 to 1000 µm. The bandwidth obtained was 30 kHz with voltage compliance ± 15 V. The neurostimulator can be easily scaled up using the provided in/out trigger ports and adapted to other applications with minor modifications.

**Graphical Abstract:**

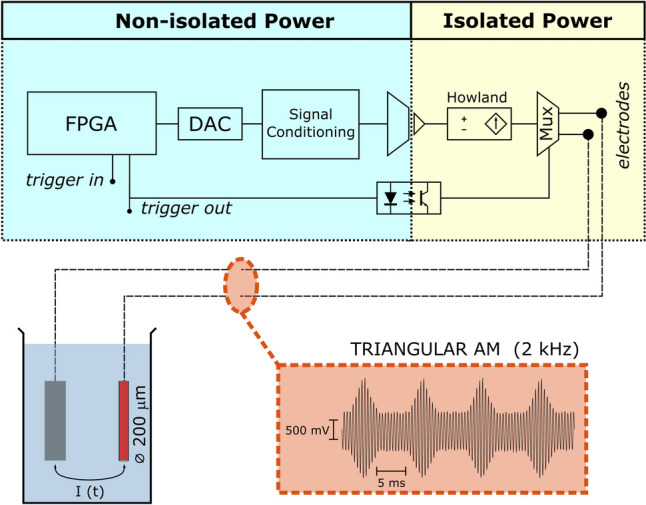

**Supplementary Information:**

The online version contains supplementary material available at 10.1007/s11517-024-03241-6.

## Introduction

The delivery of electric current to elicit neural responses has been used to treat a number of medical conditions. For example, the cochlear implant (also known as the bionic ear) [1] allows the deaf to recover functional audition by transforming acoustic signals into a series of electric pulses that are delivered to the target neurons in the inner ear. An electrode array is implanted in the cochlea in close proximity to the bipolar ganglion cells [2]. By activating these cells, the bionic ear can send neural messages to the brain thus eliciting auditory sensations [3–5]. In the same lines, visual prostheses aim to restore sight by stimulating the neurons in the retina [6, 7], the optic nerve [8], or directly in the visual cortex [9]. However, the applications of neural stimulation are not limited to restoring different sensory modalities. Among others, deep brain stimulation has been used to successfully treat Parkinson’s disease [10, 11], spinal cord stimulation can provide pain relief [12, 13], and percutaneous tibial nerve electrostimulation can assist with providing a therapy for fecal incontinence [14]; these are just a few examples of the myriad applications of electrical neuromodulation.

The delivery of electric current to the target tissue requires the use of electrodes, typically made of metals such as platinum, iridium, or titanium. Charge transfer takes place during the course of the electrochemical reactions that occur at the electrode-tissue interface. To avoid damaging the electrodes, the charge thus delivered to activate the underlying neurons has to be quickly recovered. In doing so, standard neurostimulation has relied on the use of biphasic constant-current pulses that ensure undesired electrochemical reactions do not occur [15]. Furthermore, these waveforms can be easily generated using CMOS current source-sink pairs [16, 17]. Nevertheless, recent research advances indicate that more sophisticated stimulation waveforms may be required to substantially improve the effectiveness of electrostimulation therapies. Grossman et al. [18] have successfully recruited neurons, deep in the brain, by creating interfering patterns using two high-frequency sinusoidal waveforms delivered at the surface of the skull. Twyford et al. [19] have shown that preferential activation of the ON- and OFF- retinal ganglion cells can be achieved by modulating the amplitude of high-count pulse trains, and Corna and co-workers have reported robust localized activation of retinal neurons by means of time-continuous electrical stimulation [20]. Furthermore, given the upswing of the electroceutical industry, a discipline that aims to replace some of the pharmacological therapies by selectively activating the nerve fibers [21], new waveforms that enable for more selective recruitment of the neural targets are now needed [22].

In this context and prior to bringing new solutions into the neurostimulation market, further research has to be conducted to demonstrate the potential of the emerging therapies. However, the scientific community is currently lacking accessible neural stimulators able to deliver arbitrary current waveforms during pre-clinical investigations. Some neurostimulators reported in the literature require application specific integrated circuits (ASICs) that may not be easily available [23, 24]. This article presents a programmable system and a circuit designed from of-the-shelf open-source elements and basic electronic components to deliver two electrically isolated arbitrary waveforms during experimental research. This work aims to assist the scientific community by sharing the design of an affordable and multipurpose neural stimulator able to deliver arbitrary current waveforms to different types of electrodes without the need for designing an ASIC.

## System requirements

### Targets for neural excitation

Electrical stimulation of the neurons pursues to elicit action potentials by delivering controlled electric current. From spinal cord neuromodulation [13, 25] to the bionic ear [3, 5], recruitment of the diverse neural targets has helped treating disease and restoring body function. Each neurostimulation modality requires a particular electrode type and configuration, benefits from different stimulating strategies, and exhibits its own typical activation threshold. This system has been designed to drive a broad spectrum of neural targets in the laboratory, with a focus on sensory prostheses such as suprachoroidal retinal implants [26–28]. Additionally, the system can be adapted to be used in research scenarios where small brain structures are stimulated using fine microwire electrodes [25, 29, 30]. This device allows for investigating the benefits of a number of stimulation strategies that include current steering [31, 32], temporal interference [18], and stochastic facilitation [33] among others. Thus, a minimum of two electrically isolated channels are required, able to drive electrodes with diameters ranging from few millimeters to tens of microns. Along these lines, the system presented herein can be easily scaled up to drive several electrodes simultaneously.

### Current and voltage requirements

Perhaps, the most important design requirement of a neural stimulator is that it must be capable of delivering sufficient current in order to activate the neural targets. The minimum amount of current required to elicit a neural response is given by the activation threshold of the target tissue. This threshold depends on the particular type of neuron, the size of the electrode, the physiological medium, and the distance between the electrode and the target. Thus, there is a body of literature on the activation thresholds required by retinal prostheses. In particular, experimental suprachoroidal electrodes are placed relatively far (few hundred microns) from the retinal ganglion cells, that is, the neural targets [26]. Typically, the current densities that elicit neural activation range from less than 100 µC·cm^−2^ (< 400 µm in diameter) [26–28] to around 10 µC·cm^−2^ (2 mm in diameter) [34, 35] for platinum electrodes, which represents maximum current levels of 2.4 mA. At the other extreme, brain stimulation can be achieved using low current levels by placing very small electrodes in intimate proximity to the target neurons. While activation thresholds reported for tungsten microwires range from approximately 80 µA for diameters of 50 µm [25] to up to 1 mA for electrodes 200 µm in diameter [36], for this work, we focus on relatively large electrodes (in the range of few hundreds of microns) as those employed in retinal prostheses. On the one hand, larger electrodes present lower impedances (10–50 k Ω) [37] and need to be driven with currents that do not exceed 3 mA to achieve good neural responses. On the other hand, the impedance of microwire electrodes is typically in the range 100–500 k Ω [38], and therefore they require current levels below 1 mA to successfully recruit the neural targets. The neurostimulator described here was designed to allow driving relatively low impedance electrodes (10–15 k Ω) with high current levels (~ 1 mA). Thus, the stimulus supply voltage (V_s_) must exceed the voltage drop at the electrode plus the voltage across the current source, that is, V_s_ ≥ ± 15 v. 

### Bandwidth

High-frequency electrostimulation is being applied to treat many medical conditions. For example, preferential activation of different visual pathways has been reported using electric current with frequencies comprised between 2 and 6.25 kHz [39]. Nerve conduction blockade can be achieved by delivering current at 10 kHz at the spinal cord for back pain management [13]. In the same range of frequencies, non-invasive deep brain electrostimulation has been reported by creating interfering patterns of high-frequency sinusoidal current waveforms [18]. Some of these therapies rely on the use of constant-current pulses. These pulses have been classically used in a wide spectrum of neurostimulation scenarios, typically of two symmetrical phases with the charge balanced. The phase time of these pulses ranges from few milliseconds, typically delivered via large electrodes, to few tens of microseconds for small microwire electrodes. Thus, the minimum sampling rate required to reconstruct a 50 µs pulse will be 40 kHz, that is, one sample every 25 µs. However, the delivery of some therapies may require the use of shorter pulses, as in the case of preferential retinal neuromodulation [39] or the treatment of chronic back pain [13], where the duration of the pulses reported were 30 µs and 40 µs respectively. Thus, to allow further investigation of these stimulation paradigms, a sampling time of between 15 and 20 µs is needed, that is, a sampling rate of approximately 60 kHz. Considering the reconstruction limit defined by Nyquist’s theorem, this design requirement will enable the generation of arbitrary current waveforms with bandwidths up to 30 kHz. 

### Electrical isolation

Good electrical isolation is essential to ensure that all current delivered through the stimulation electrode is recovered via the designated return electrode. This is not only a safety requirement but also a must to ensure scientific validity of the experiments, as leaking current might activate undesired neural targets. A minimum of continuous isolation voltage of 1000 VDC will be required at any node of the stimulator circuit wired to the electrodes, including power supply and any input/output of the system.

### Avoiding charge accumulation

The irreversible electrochemical reactions that can occur at the electro-tissue interface define the limits of charge delivery for metallic electrodes [40]. Charge-balanced constant-current pulses below said limits allow recovering the charge delivered to elicit a neural response during the second phase of the stimulus [41]. However, with the advent of arbitrary current waveforms, the user must ensure that the charge thus supplied does not damage the electrodes or the tissue by carefully designing the current waveforms. In addition, the electrodes are to be disconnected from the stimulator when unused [17].

### Usability

The neurostimulator described here has been conceived to be used in preclinical scenarios for the investigation of a broad repertoire of neural phenomena including the selective activation of retinal ganglion cell types using kilo-hertz electrical stimulation in visual prostheses [19, 39, 42]. Acute electrophysiology experiments often require the repetition of a set of stimuli upon arrival of a trigger signal, as in the case of closed-loop electrostimulation [43]. In other occasions, the experimenter may need the stimuli to be automatically delivered (internal trigger). At least, the system must be able to store a stimulus waveform of length up to 0.5 s and allow to quickly upload different stimuli from a computer, taking less than a second to upload the biggest possible stimulus. Assuming the stimuli are delivered at the maximum sampling rate, that is 60 kHz, using a 12-bit analog-to-digital converter (ADC), the minimum memory required is of nearly 45 Kbytes per channel. Furthermore, the device must be easily programmable, relying on a user-friendly scripting language.

## System design

**Fig. 1:**
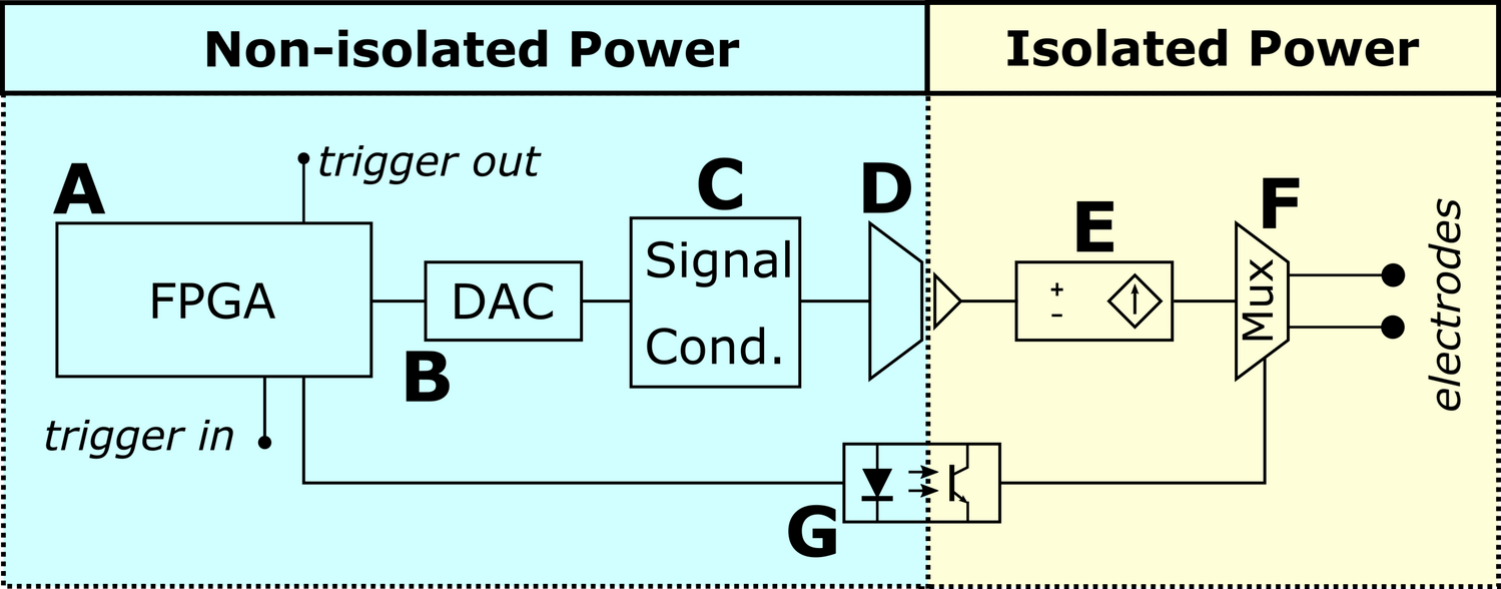
Block diagram of one channel of the arbitrary current stimulator. The left panel, highlighted in blue, describes the non-isolated subsystems. The right panel, highlighted in yellow, shows the electrically isolated subsystems. (**A**) A digital processing unit, implemented using a Field Programmable Gate Array (FPGA), that stores a set of stimuli and generates the stimulus waveforms using (**B**) a digital-to-analog converter (DAC). A signal conditioning block (**C**) provides low-pass filtering functions and offset removal. Then, an isolation amplifier (**D**) provides appropriate isolation relative to ground. A voltage-dependent current source (**E**) converts voltage waveforms into current waveforms, which are delivered to the target tissue via a pair of electrodes. To connect/disconnect the source to the electrodes, an electronic multiplexer, controlled by the FPGA via an optocoupler (**G**), is used. The system can receive/send trigger in/out signals to synchronize the delivery of the stimuli

This section describes the subsystems of a 2-channel arbitrary neurostimulator designed to meet the physiological and functional requirements previously described. A block diagram depicting the hardware components of one channel of the device is shown in Fig. 1. An FPGA device provides the system with the capability of storing the samples of a set of two large stimuli for delivery as programmed by the user. A digital-to-analog converter (DAC), connected to the FPGA, is used to generate the stimulus waveforms of each channel. The signal thus generated is first low-pass filtered and then adjusted to a symmetrical range from −3.3 to 3.3 V. After this, the signal is passed onto an isolation amplifier. Thereafter, all subsystems are powered using an isolated power supply. Next, a voltage-to-current converter generates the stimulation current waveforms, and an electronic multiplexer connects/disconnects the current source to the electrodes. In addition, the FPGA device can receive a trigger in signal or generate a trigger out signal to provide an electric token of the onset of the stimulus delivery. It must be noted that, while the FPGA supports two channels, each channel requires its own DAC and analog circuitry (signal conditioning, isolation, voltage-to-current conversion, and electrode shorting) and thus serves an independent pair of electrodes.

### Digital subsystem

#### iCEBreaker FPGA board

The control logic for the neurostimulator presented here has been implemented in an FPGA. The decision of whether to use an FPGA or a microprocessor platform is not easy in this case. On the one hand, microprocessors have several advantages: They are generally cheaper and easier to program. On the other hand, they lack precise time control as sample settle time may depend on the actual pathways taken during the execution of the code. FPGAs, however, are becoming cheaper, and better firmware development tools are continuously released by the manufacturers and the open-source FPGA tooling community. With an FPGA, we have cycle-accurate precise control over timing, which can be easily made independent of the specific circuit workload. Furthermore, the verification strategy available for FPGA code facilitates a roadmap to the clinic as a medical device, as strict regulatory compliance requirements can be more predictably achieved due to lack of elements of unknown provenance such as third-party libraries and issues related to operating systems, for example, the effects of multiprocessing or memory allocation on task scheduling and execution, among others. Furthermore, the FPGA technology offers substantial possibilities for scalability, for example, given a device with more resources, more channels can be easily added, without any problems of timing, synchronization, or computing power.

Lattice iCE40 FPGAs are inexpensive, low-power consuming and easily available, and therefore represent an excellent option to meet some of the requirements of the neurostimulator described here. An iCEBreaker FPGA board, Version 1.0c [44], was chosen as it is well documented, popular, open-source, and well supported by an extensive community thus providing an excellent scalable platform. Furthermore, the FPGA board is supported by the open-source FPGA tools. In particular, this design has been implemented using ghdl [45], ghdl-yosys-plugin [46], yosys [47], nextpnr [47], and icestorm [48]. Synthesis is performed with yosys (Yosys Open SYnthesis Suite), a configurable synthesis tool that can map its synthesis results to Lattice iCE40 FPGAs, among others, but since the open-source version of yosys only supports the Verilog hardware description language, ghdl and ghdl-yosys-plugin have been used as a VHDL front-end for the synthesis tool. The capabilities of the ghdl VHDL simulator are extended by the ghdl-yosys-plugin, which is a plugin for yosys that, based on the features provided by ghdl, provides a front-end capable of reading and synthesizing VHDL files. Nextpnr —a timing-driven place and route tool that can be adapted to different FPGA families— performs the placement and routing of the design, whereas icestorm, a set of tools to analyze and create bitstreams of the Lattice iCE40 FPGA family, is used both to generate the FPGA configuration files and to download them into the iCEBreaker FPGA board. The iCEBreaker FPGA board assembles a Lattice iCE40UP5K FPGA from the iCE40 UltraPlus Family, a device that includes 5280 logic cells (each one with a 4-input Look-up-Table, a single Flip-flop, and carry logic); 30 Kbits of dual-port RAMs; 1 Mbit of single-port RAM; 8 DSP blocks; and hard IP cores such as I2C, SPI, and PWM, among others.

#### FPGA design architecture: icestim

**Fig. 2:**
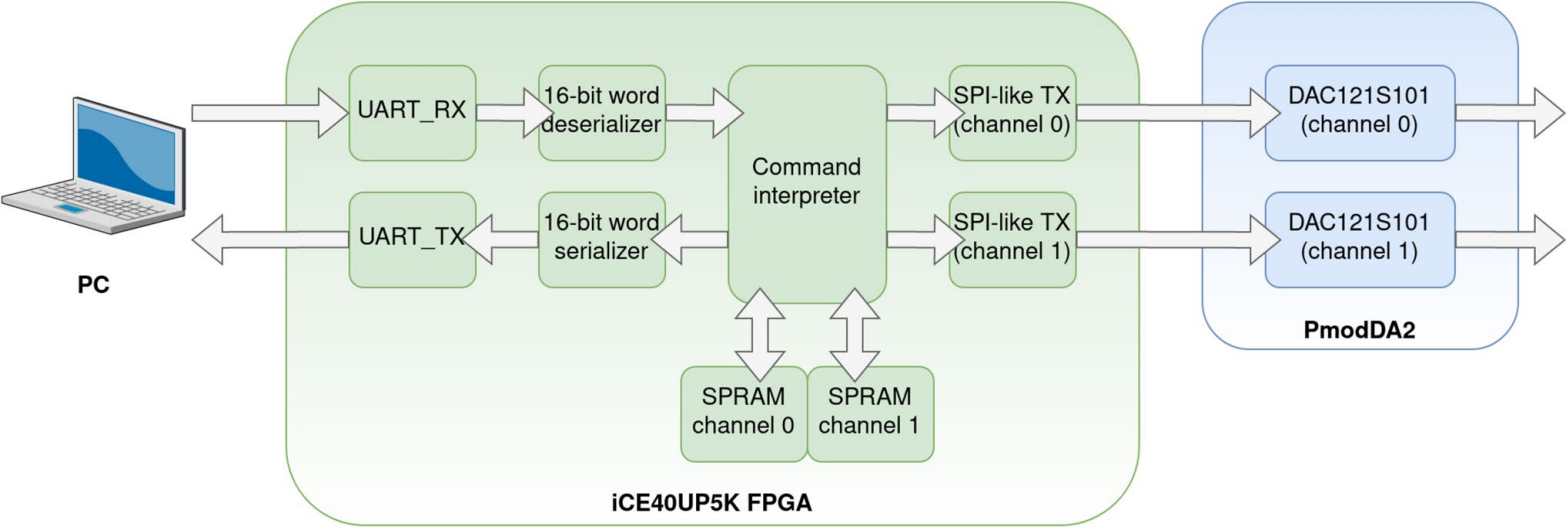
Architecture of the FPGA implementation. The PC runs the user software that communicates with the FPGA. The UART transmitter and receiver manage communications with the PC. The 16-bit word serializer and deserializer convert between the 8-bit UART protocol and the internal command representation that the design uses. The command interpreter receives 16-bit commands and operates with them as specified in the command set. The SPI-like transmitters send data to the Digital-to-Analog Converters (DACs), while the internal SPRAMs act as storage for the input stimuli

All the logic of the stimulator has been implemented for the ice40-up5k device inside the iCEBreaker FPGA board. The implemented design has been named “icestim.” An overview of the architecture of the design can be found in Fig. 2. The design contains the following functional elements:

- An UART receiver to receive data and commands from a PC.
- An UART transmitter to send data back to the PC, which can be used for debugging purposes.
- All the 4 instances of the SB_SPRAM256KA of the ice40-up5k FPGA. Since each SPRAM can store 2^14^ data of 16-bit width each, by using two SPRAMs for each channel, we have a maximum capacity of 32 k samples per channel.
- Two SPI-like transmitters to send the stimuli to the DAC module which will generate the voltage inputs for the voltage-to-current converter. The protocol is a modified version of the SPI which allows a single transmission mode and 16-bit data words.
- A 16-bit word deserializer and a 16-bit word serializer. Since the command and data are 16-bit each, but the UART protocol sends one byte at a time, the deserializer creates 16-bit words that can be processed by the system logic, whereas the serializer converts each 16-bit output of the system logic to a couple of UART transactions. The 8 least significant bits of each word are always sent first.
- A finite state machine (FSM) that implements the necessary command set to create a flexible neurostimulation platform, which is described in the following subsection.

#### Command set

**Fig. 3:**
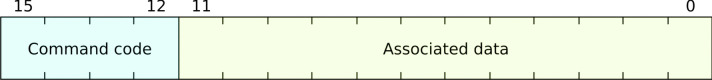
Bitfields of a 16-bit command word. The 4 MSB (bits 15 to 12) correspond to the command code, while the 12 LSB (bits 11 to 0) correspond to its associated data

16-Bit commands are sent from the PC to the configured FPGA device. As stated before, this is achieved by sending two bytes through the USB UART for each 16-bit word, from which the 8 least significant bits must be sent first. After the deserializer reconstructs the command, it is interpreted as shown in Fig. 3. The command code is located in the 4 most significant bits (MSB) of the command, whereas its associated data is located in the 12 least significant bits (LSB) of the 16-bit word. Table 1 contains the interpretation of each command in the command set.

**Table 1:** Icestim command set. The associated data are interpreted differently according to the specific command code

Command code	Command code (hex)	Functionality	Interpretation of associated data (12 LSB)
ISDATA0	0	Store data for channel 0	A single sample of the stimulus for channel 0. It is stored on the relevant position in the internal SPRAM
ISDATA1	1	Store data for channel 1	A single sample of the stimulus for channel 1. It is stored on the relevant position in the internal SPRAM
SET_SIZE_LO	2	Set size of stimuli (lowest bits)	The lowest 12 bits of the size, in samples, of the stimulus signal. It is stored it in the FSM’s internal state context
SET_SIZE_HI	3	Set size of stimuli (highest bits)	The highest 12 bits of the size, in samples, of the stimulus signal. It is stored it in the FSM’s internal state context
SET_FREQ	4	Set frequency of stimulation	Index of possible stimulation frequencies. 0 = 30 MHz, 1 = 50 MHz, 2 = 60 MHz. Other frequencies can be added easily if needed
SET_LOOPED	5	Configure stimuli to run only once or repeat continuously	0 = stimuli run only once 1 = stimuli run continuously
START	6	Begin stimulation	Ignored
PAUSE	7	Pause stimulation, if running. After the next START, it will continue from the sample where it stopped	Ignored
REWIND	8	Move the signal pointer to the first sample. Used in combination with PAUSE to restart the stimulation	Ignored
WR_REWIND	9	Move the write pointers to the first sample. Used in combination with the ISDATA0 and ISDATA1 commands to rewrite the stimuli	Ignored
UNDEFINED	A	Reserved for future expansion	Ignored
DBG_ECHO	B	Send back to the PC the same 16 bits that were received. Implemented for debugging purposes	Sent back to the PC with the command code
DBG_READ	C	Read a single value from the stimulator context, and send it back to the PC	Will send back to the PC a different value to according to the value of the associated data (hex): 0 = SIZE_LO (12 LSB of stimuli size) 1 = SIZE_HI (12 MSB of stimuli size) 2 = FREQ (frequency) 3 = CYCLE_COUNT (internal counter that implements temporal separation of the samples according to the configured frequency) 4 = RUNNING (whether the stimulator is running) 5 = LOOPED (whether the simulator will run continuously or not) 6 = RD_PTR_LO (lowest 12 LSB of the signal pointer) 7 = RD_PTR_HI (highest 12 LSB of the signal pointer) 8 = WR_PTR0_LO (lowest 12 LSB of the write pointer for channel 0) 9 = WR_PTR0_HI (highest 12 LSB of the write pointer for channel 0) A = WR_PTR1_LO (lowest 12 LSB of the write pointer for channel 1) B = WR_PTR1_HI (highest 12 LSB of the write pointer for channel 1)
DBG_DUMP0	D	Read all stimuli stored for channel 0. Stops any ongoing stimulation and will rewind the vectors after finishing	Ignored
DBG_DUMP1	E	Read all stimuli stored for channel 1. Stops any ongoing stimulation and will rewind the vectors after finishing	Ignored
NOP	F	Do nothing	Ignored

### Arbitrary analog signal generation

**Fig. 4:**
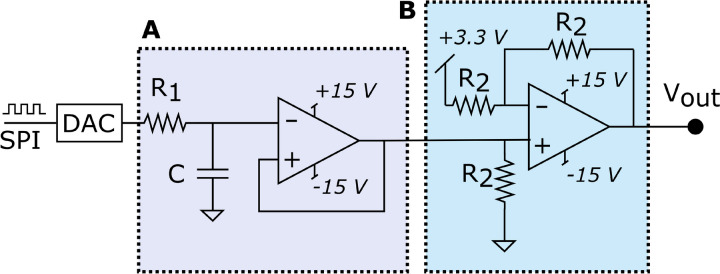
Arbitrary analog voltage generation stage. The digital outputs from the FPGA are converted to analog voltages by a digital-to-analog converter. The analog voltage then passes through **A** a low-pass filter that acts as a reconstruction filter, smoothing the signal, and **B** a subtracting amplifier that centers the signal around 0 V. The values chosen for the individual components are R1 = 2,2 k Ω, C = 2,2 nF, and R2 = 1 k Ω. The two operational amplifiers included in the TL072ACP (Texas Instruments, Dallas, USA) chip were used in this subsystem

The architecture of the arbitrary analog voltage generation stage is summarized in Fig. 4. The design uses Digilent’s PmodDA2 to generate the analog outputs. This PMOD (Peripheral Module) contains two DAC121S101 digital-to-analog converters, one for each channel. Each of those DACs functions as a zero-order hold for the purpose of signal reconstruction. Each DAC uses an SPI-compatible protocol to receive data, which corresponds to an SPI particularized to the following parameters: polarity = false, phase = true, word size = 16 bit, and bit order = MSB first. Each SPI transmission corresponds to a single 12-bit sample of an input stimulus. Subsequently, the analog output of the DAC is low-pass filtered using an RC active filter with cut-off frequency set to 33 kHz based on Nyquist’s theorem. Next, we used a voltage amplifier and substractor to center the analog signal around zero between −3.3 and 3.3 V.

### Electrical isolation

To provide electrical isolation from ground, arbitrary waveforms generated as described previously were passed through a wideband isolation amplifier AD215AY (Analog Devices, Massachusetts, USA). Likewise, digital triggers were also electrically isolated via optocouplers. All electronic subsystems beyond the isolation amplifier were powered using DC-DC isolated converters. Thus, the isolation amplifier and the operational amplifiers used in the voltage-to-current conversion stage were powered using the 2233641 (RS PRO, London, UK) DC-DC converters. The optocoupler was powered using the ITA0505S (XP Power, Singapore) DC-DC. It must be noted that each channel’s circuitry is powered by isolated DC/DC converters and therefore channels work with independent grounds.

### Voltage-to-current converter

**Fig. 5:**
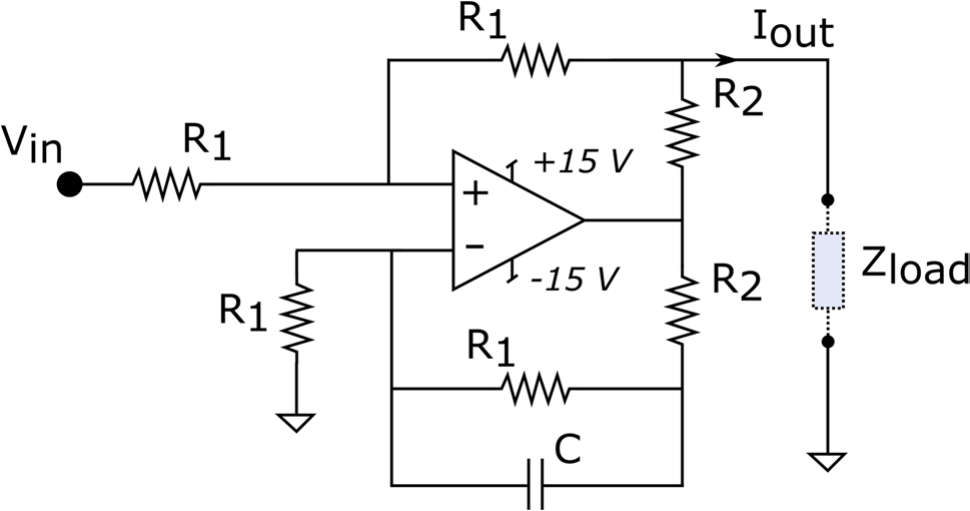
Circuit schematic of a modified Howland voltage-to-current converter using an operational amplifier TL072ACP. In the system described herein, the values of the individual components were R1 = 300 k Ω, R2 = 1 k Ω and C = 3pF. V_in_ denotes the voltage waveform from the isolation amplifier, and Z_load_ is the equivalent impedance of the electrodes connected to the stimulator driven by the output current I_out_. This Z_load_ represents the impedance of the electrode-tissue-electrode interface seen by the pair of electrodes (stimulus and return). The multiplexer connected between the output and the load has not been illustrated for simplicity

In this work, the current driver is implemented by a voltage-to-current converter. This subsystem is connected to the electrodes through electronic switches and therefore represents a critical element in the design of the neural stimulator, as this circuit is responsible for the delivery of charge to the target tissue. A Howland current source was chosen as it is characterized by high output impedance, a wide bandwidth, and can deliver accurately arbitrary waveforms [49]. Different topologies also exist [50] that allow for extending the bandwidth beyond the target of 30 kHz required for neural stimulation to up to 200 kHz while keeping the output impedance above 3 M Ω. Figure 5 shows a circuit diagram of the topology of the current source used in this system.

### Stimulus triggering

**Fig. 6:**
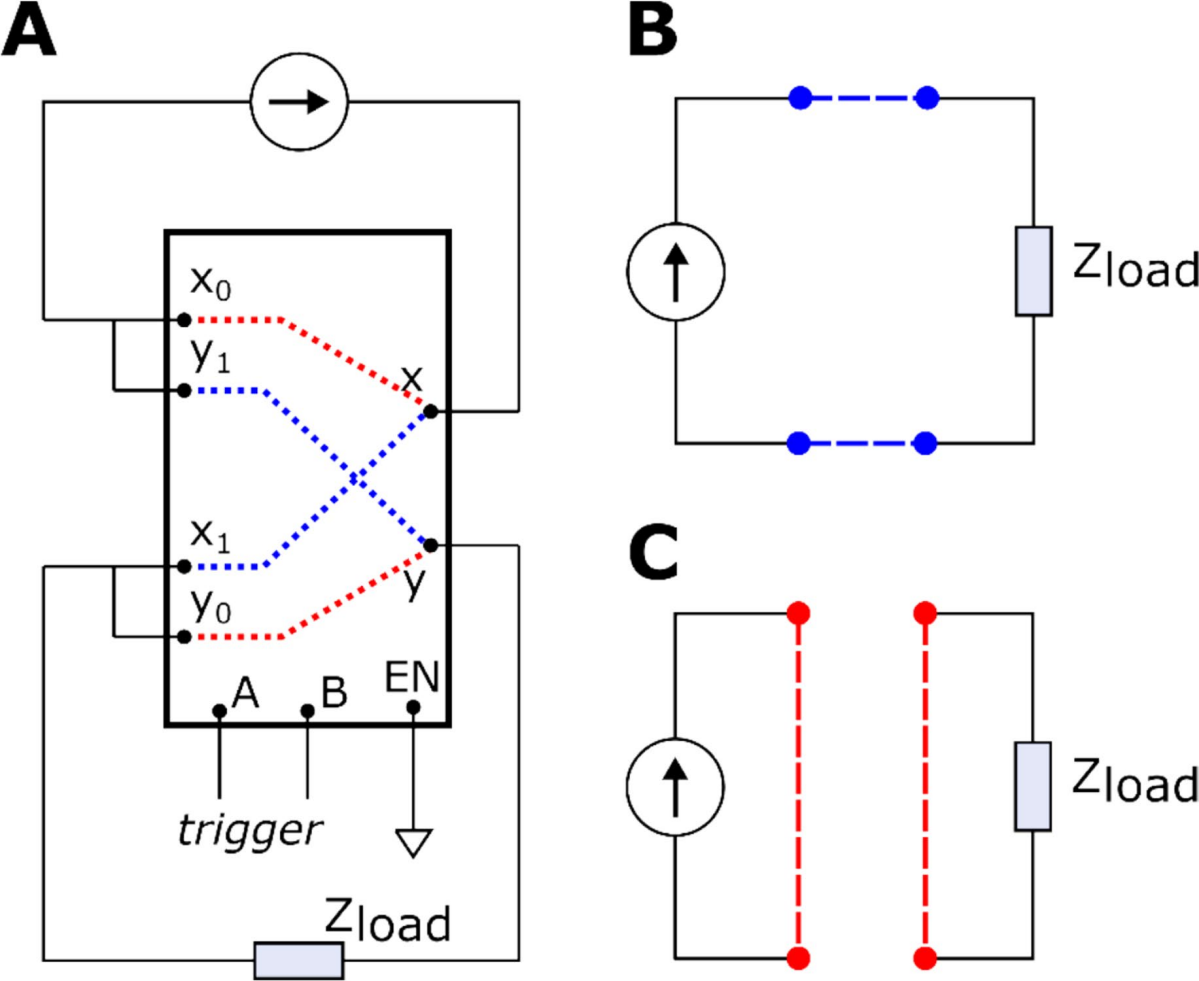
**A** A diagram illustrating the wiring of the multiplexor CD4053BE. The current source represents the output of the Howland voltage-to-current converter, and Z_load_ is the target electrode. A differential trigger signal is to be used between terminals A and B of the chip to modify the internal connections. Blue and red dotted lines represent the internal switching for ON and OFF trigger logic respectively. **B** The resulting wiring with trigger ON. The neurostimulator is connected to the electrode. **C** exemplifies the resulting wiring with the trigger OFF. Both the current source and the load are shorted

The trigger signal in the isolated analog subsystem is used to provide a time reference for the stimulus delivery and to connect and disconnect the current source to the electrode. When the trigger is asserted, the current source is effectively connected to the electrode. On trigger deassertion, the current source is disconnected from the electrode. This disconnection is achieved by both shorting the terminals of the electrode and shorting the output terminals of the current source. To implement this shorting, a CD4053BE (Texas Instruments, Dallas, USA) digitally controlled analog multiplexer has been used, as shown in Fig. 6. Note that the trigger signal can be generated from the FPGA subsystem or, alternatively, from an external source.

### Stimulation control

A python script to control the stimulation was developed, which receives two .csv files that contain the stimuli (one per channel) and optionally the --freq and --looped arguments, which allow to set the stimulation frequency and stimuli looping, respectively. As it will be shown below, the python script makes many data consistency checks, so any issues with the input data, the design or its operation are quickly found. The python script that controls the stimulation and an additional GNU Octave script to generate two sample waveforms, one for each channel, have been added as supplementary materials.

**Fig. 7:**
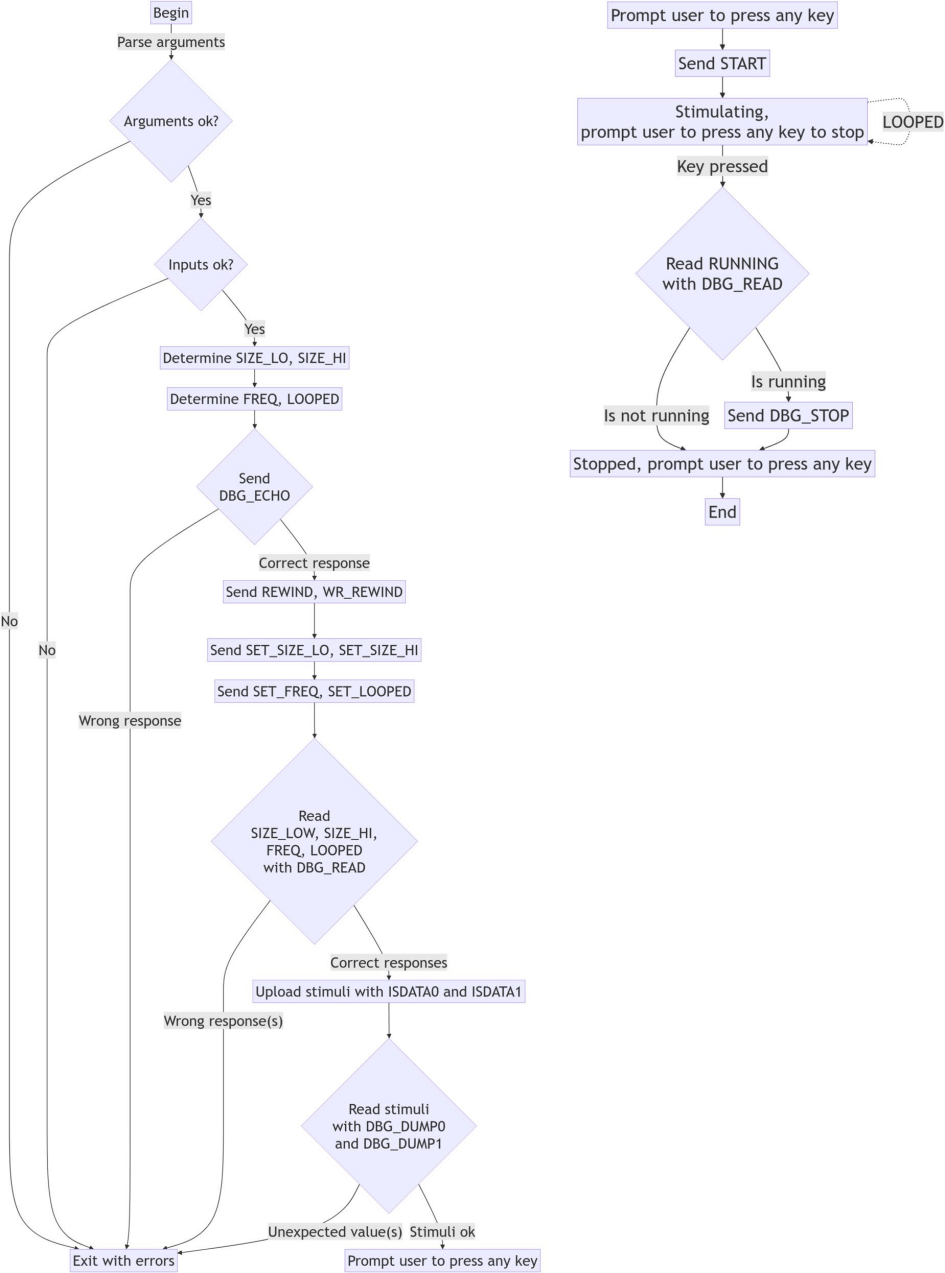
Simplified flow diagram of the stimulation control script. The left side shows all initialization checks and stimuli loading and verifying, while the right side shows the actual stimuli execution

Figure 7 shows a simplified flow diagram of the simulation control script. The script performs the following duties:

- Parse and sanitize the input stimuli for both channels. Since we are working with a 12-bit DAC, each sample must have an integer value between -2048 and 2047. Also, stimuli length (in number of samples) is checked to ensure both stimuli have the same length. Finally, the length of the stimuli is checked against the maximum capacity in the FPGA. If any issues are found with the input stimuli, an error is reported and the script exits.
- The SIZE_LO and SIZE_HI parameters are determined from the stimuli length, and FREQ and LOOP parameters are determined from the input arguments (if these last two are not set, default values are used).
- A DBG_ECHO command is sent to the FPGA device to ensure that it has been correctly configured. If the response to the command is not correct, an error is reported and the script exits.
- The signal and write pointers are rewinded with the REWIND and WR_REWIND commands, respectively. While this is not needed if the FPGA bitstream has just been downloaded, this is required any time we want to rewrite the stimuli on an already-configured FPGA.
- The stimuli size is configured by sending the SET_SIZE_LO and SET_SIZE_HI commands.
- Frequency and stimuli repetition are configured by sending the SET_FREQ and SET_LOOP commands.
- A number of DBG_READ commands are issued to confirm the design was correctly configured. SIZE_LO, SIZE_HI, FREQ, and LOOPED are read from the FPGA and compared with the expected values. If any of the read values does not match its expected value (meaning the correct value determined by the script, which is the one that was sent to the FPGA), an error is reported and the script exits.
- Stimuli are sent with multiple ISDATA0 and ISDATA1 commands, one per stimuli sample. It must be noted that, with the UART interface configured at 1 Mbaud, this data transfer occurs at 100 Kbytes per second, and thus it will take less than a second even for the longest stimulus that can fit inside the FPGA memory.
- The uploaded stimuli are verified by issuing the DBG_DUMP0 and DBG_DUMP1 commands. If any stimuli sample read from the FPGA differs from the stimuli sent by the PC, an error is reported and the script exits.
- The user is prompted to press any key to send the START command. When a key is pressed, the START command is sent, and the stimulation begins.
- The user is prompted to press any key to send the PAUSE command. When a key is pressed, the script will first use the DBG_READ to check whether the stimulator is running, and in case it is running, it will send the PAUSE command.
- The user is prompted to press any key to exit the script.

### Device operation

In order to use the stimulator, the user only needs to generate two .csv files; each one will contain the stimulus for a single channel. The format for the .csv files is fairly simple: Each line contains a single sample, which takes an integer value between -2048 and 2047. These correspond to the available range in a 12-bit signed number. These can be easily generated, using GNU Octave or Matlab, from an already existing signal, just by scaling it to the aforementioned range, quantizing it to integer values, and writing it to a file.

After the .csv files are generated, the user just needs to invoke the stimulation script by executing the stimulation control script with the appropriate arguments:

python3 stimulate.py input0.csv input1.csv --freq 60 --looped

## Characterization and results

### Design verification of the digital subsystem

**Fig. 8:**
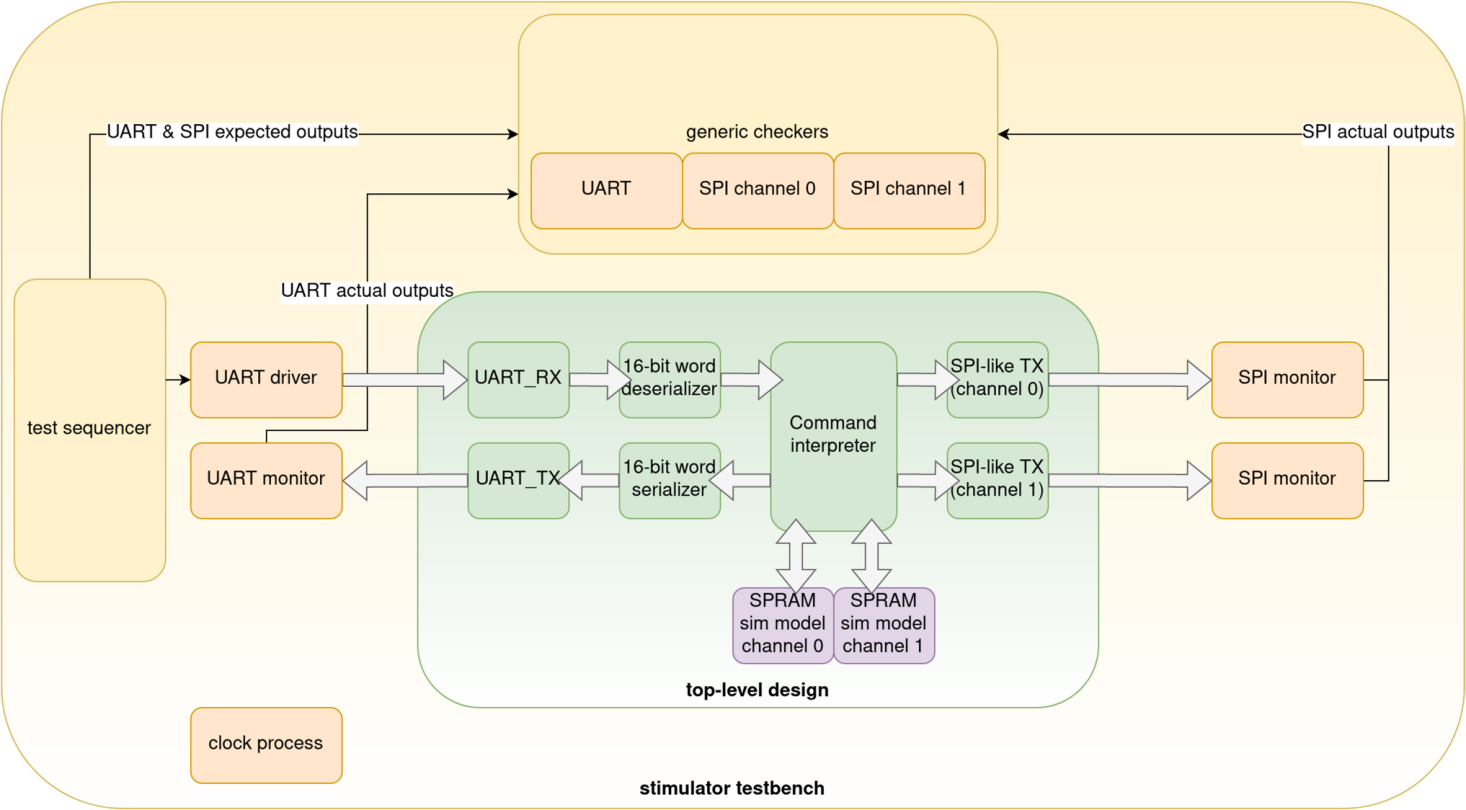
Testbench architecture for the verification of the FPGA design. The testbench is structured as a TLM (transaction-level model) testbench, meaning that design inputs and outputs are separated in two different abstraction levels; at the transaction level, only the data that moves through the interfaces is considered, whereas at the pin level, all signal movements are taken into account. The test sequencer generates high-level transactions that are converted by the UART driver into sequences of pin movements. The UART and SPI monitors are constantly monitoring the design outputs to check for correctness of the specific protocol such as correct bit times or transitions, among others. The outputs of the monitors are transactions that only contain the information about the specific user data that is being propagated through the interface. This way, the generic checkers only have to check that the actual output values match the expected output values, since the output waveforms are being verified by the protocol monitors

Design verification was done both in simulation and over the configured FPGA design. A testbench for the stimulator was first developed, so the command set could be tested in simulation. The testbench architecture can be found in Fig. 8. Since the SPRAMs are black-boxes implemented in silicon in the FPGA device, a simulation model for the SPRAM was developed for the testbench.

Several tests were written for the commands in the command set. Since a typical stimulation requires multiple commands, for this specific design, it is not convenient to write a test for each command in isolation. Thus, all tests involve multiple commands of the command set. Each test sends a number of high-level transactions (in this case, 16-bit commands) to the design under test and expects to see transactions with specific values at the output ports of the design (in this case, the UART output and the two SPI-like outputs for the stimulation channels). See Table 2 for the exact relation of what commands are involved in each of the tests.

**Table 2:** Simulation tests for the FPGA implementation

Test name	Summary	Commands involved
Test_ISDATA0	Send stimuli data for channel 0, check the write pointer is incrementing correctly, and check that stimuli was stored in the SPRAM correctly	SET_SIZE_LO, SET_SIZE_HI, ISDATA0, DBG_READ, DBG_DUMP0
Test_ISDATA1	Send stimuli data for channel 1, check the write pointer is incrementing correctly, and check that stimuli was stored in the SPRAM correctly	SET_SIZE_LO, SET_SIZE_HI, ISDATA1, DBG_READ, DBG_DUMP1
Test_SET_SIZE_LO	Write the lowest 12 bits of the stimuli size and check they were stored correctly	SET_SIZE_LO, DBG_READ
Test_SET_SIZE_HI	Write the highest 12 bits of the stimuli size and check they were stored correctly	SET_SIZE_HI, DBG_READ
Test_SET_FREQ	Set the frequency and check it is stored correctly	SET_FREQ, DBG_READ
Test_SET_LOOPED	Set the LOOPED variable and check it is stored correctly	SET_LOOPED, DBG_READ
Test_START	Write stimuli for the two channels, begin the stimulation, and check the stimuli are correctly reproduced in the output SPI-like ports	SET_SIZE_LO, SET_SIZE_HI, ISDATA0, ISDATA1, DBG_READ, DBG_DUMP0, START
Test_LOOPED	Write stimuli for channel 0, configure stimulator to loop stimuli, begin the stimulation, and check the stimuli appear multiple times in the corresponding output port	SET_SIZE_LO, SET_SIZE_HI, SET_FREQ, SET_LOOPED, ISDATA0, START
Test_PAUSE	Write stimuli for channel 0, begin the stimulation, pause the stimulation before it finishes, read the signal pointer and check it matches the expected value, resume the stimulation, all this while checking the correctness of the outputs	SET_SIZE_LO, SET_SIZE_HI, ISDATA0, DBG_READ, START, PAUSE
Test_REWIND	Write stimuli for channel 0, begin stimulation, wait until it ends, read the signal pointer, rewind the signal pointer and read it again	SET_SIZE_LO, SET_SIZE_HI, ISDATA0, START, DBG_READ, PAUSE, REWIND
Test_WR_REWIND	Write stimuli for channel 0, read the write pointer, rewind the write pointer and read it again	SET_SIZE_LO, SET_SIZE_HI, ISDATA0, DBG_READ, WR_REWIND
Test_DBG_ECHO	Send some data, check that it is echoed back correctly	DBG_ECHO

The design was simulated with the open-source simulator GHDL version 4.0.0-dev (3.0.0.r329.g63eee3d6b). The open-source VUnit test framework [51], version 4.7.0, was used to manage and run the tests. Line coverage reported by GHDL was 91.4% for the synthesizable sources.

The design was implemented with the following versions of the previously mentioned open-source tools: GHDL (version 4.0.0-dev (3.0.0.r329.g63eee3d6b)), ghdl-yosys-plugin (commit 5b64ccf) and Yosys (version0.30 + 48) for synthesis, nextpnr (version nextpnr-0.6–29-g54b20457) for place and route, and icestorm (commit d20a5e9) for bitstream generation and FPGA configuration.

### Bench testing and characterization

In order to provide an initial validation of the gain of the device, meaning its transconductance or the output current as a function of the input voltage, we performed tests using a 1 K Ω resistor as load, since the impedance remains constant with frequency. A 2.0 V peak-to-peak sinusoidal voltage waveform with frequencies varying from 100 Hz to 30 kHz was applied. A Picoscope 2204A (Pico Technology, Cambridge, UK) oscilloscope connected to a laptop running on batteries to provide electrical isolation was used to measure the voltage across the resistor. The amplitude of the voltage signal thus measured remained approximately flat between 100 Hz and 10 kHz, decaying progressively from 1.01 ± 0.03 V at 100 Hz to 0.95 ± 0.05 V at 10 kHz. In other words, the transconductance of the voltage-to-current converter remained approximately constant in said frequency range at 1 mA·V^−1^. However, a more marked decay was observed beyond 10 kHz, decreasing to 0.75 ± 0.02 mA·V^−1^ at 30 kHz.

**Fig. 9:**
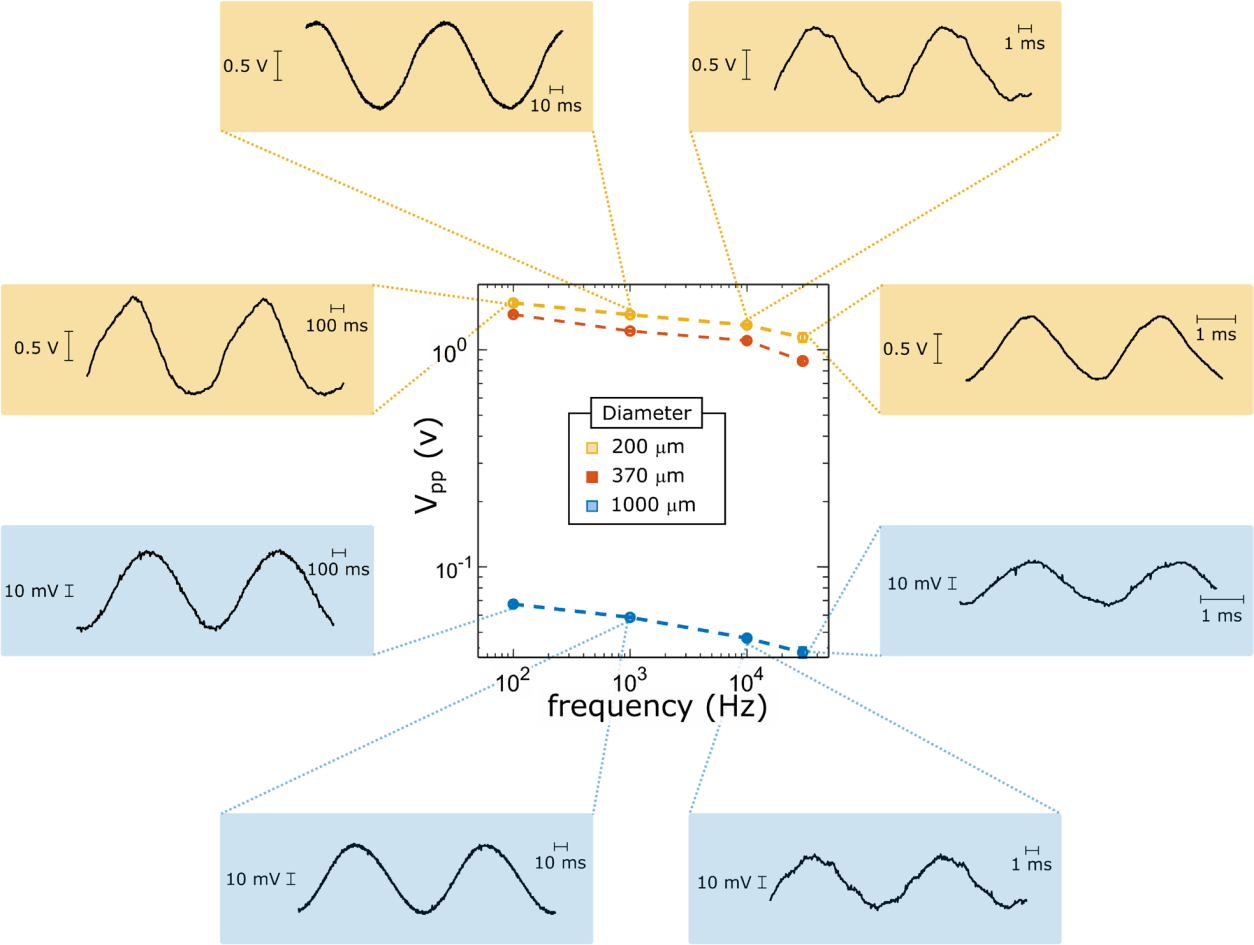
Peak-to-peak voltage (V_pp_) across electrodes driven by sinusoidal current waveforms of different frequencies. The voltage waveforms applied to the voltage-to-current converter had 2.2 V peak-to-peak amplitude, which represents a current amplitude of approximately 2.2 mA peak-to-peak

The arbitrary neural stimulator described here was also tested using custom wire electrodes with diameters of 200 and 370 µm made from silver wire (Advent Research Materials, Oxford, UK) to mimic the typical size of retinal electrodes. Additionally, a 1 mm in diameter electrode was employed to test the performance of the system with a low-impedance electrode (Advent Research Materials, Oxford, UK). These silver wires were electrically insulated using nail polish to have only the tip exposed to saline solution (0.9% NaCl). A silver rod electrode, with diameter of 1 mm, was used as a return electrode and placed at approximately 30 mm from the active electrode. Similarly, 2.2 V peak-to-peak sinusoidal waveforms at 100 Hz, 1 kHz, 10 kHz, and 30 kHz were generated to drive the electrodes with a sinusoidal current waveform 2.2 mA peak-to-peak at said frequencies. The voltage across the terminals of the electrodes was measured, as illustrated in Fig. 9. As expected, voltages across electrodes remain approximately flat and fairly within the 3-dB bandwidth range.

**Fig. 10:**
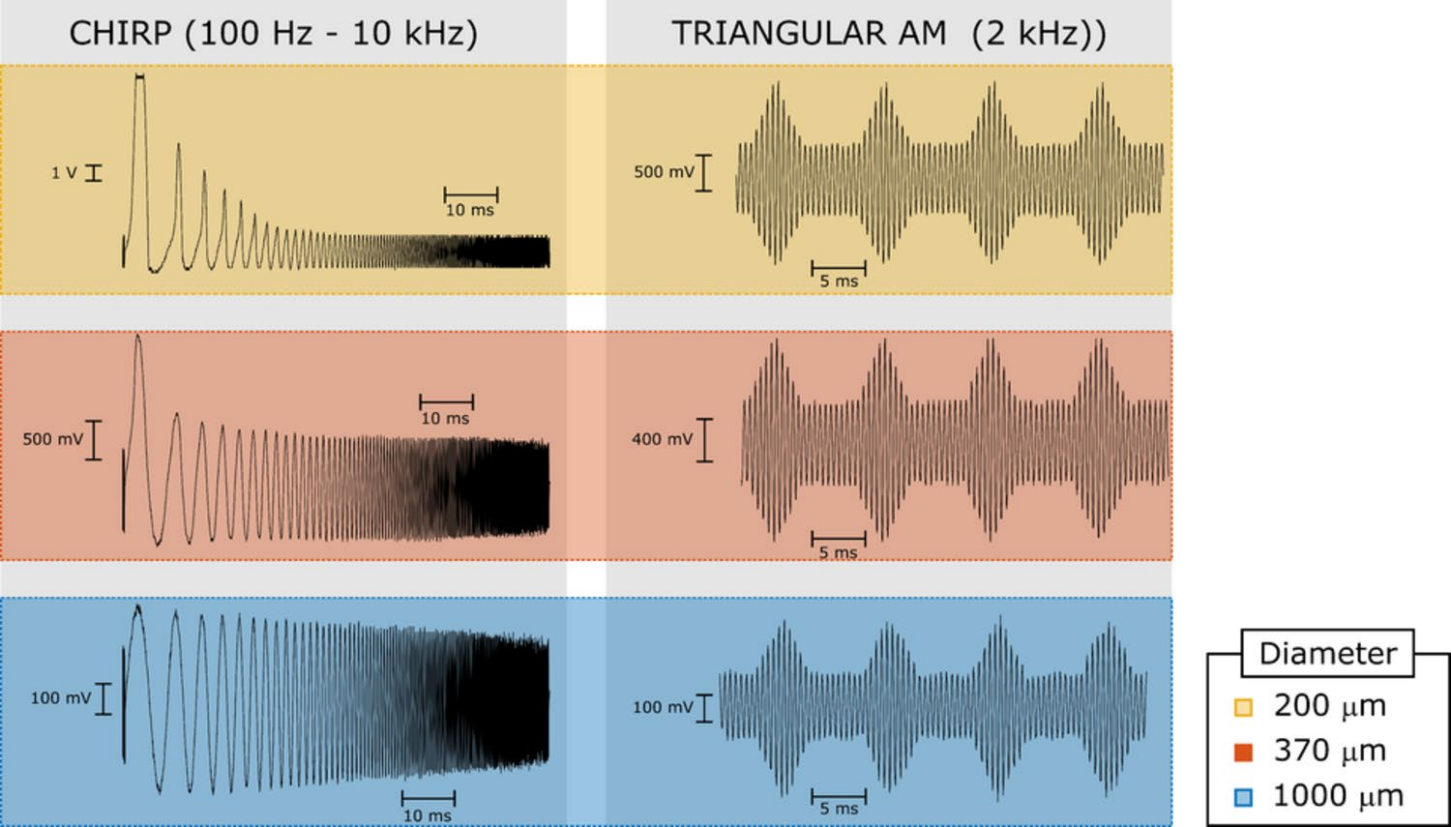
Voltage waveforms recorded across electrodes of different diameters. The panel on the left shows an 80-ms frequency sweep between 100 Hz and 10 kHz with amplitude set to 2.3 mA peak-to-peak. The panel on the right shows an amplitude-modulated sinusoidal waveform. The frequency of the carrier was 2 kHz. The envelope was a 100-Hz rectified triangular waveform with peak value equal to 0.5 and 0.5 offset

In addition, two arbitrary waveforms were generated to demonstrate the potential of the arbitrary neural stimulator described in this work. First, an 80-ms chirp waveform with frequency varying linearly between 100 Hz and 10 kHz was generated to deliver a current waveform. Secondly, a 2-kHz sinusoidal waveform with its amplitude modulated triangularly at 100 Hz was also generated to mimic the stimulation strategy employed by Twyford et al. for the preferential activation of retinal ganglion cells [19]. In both cases, amplitude was set to 2.3 mA peak-to-peak, as shown in Fig. 10. These stimuli were applied to the three types of electrodes described previously. As expected, the voltage across smaller electrodes was substantially larger than that across the larger ones, as they present higher impedance. Note that in the case of the 200-µm electrode, the stimulator saturated at lower frequencies; this is the result of a relatively large impedance at low frequencies driven by a relatively large current. Said saturation phenomenon was not observed with the 370-µm and the 1000-µm electrodes, as the voltage drop across these ones remained within the compliance limits of the voltage-to-current converter. Furthermore, all electrodes were successfully driven by the amplitude-modulated sinusoidal current waveform.

**Fig. 11:**
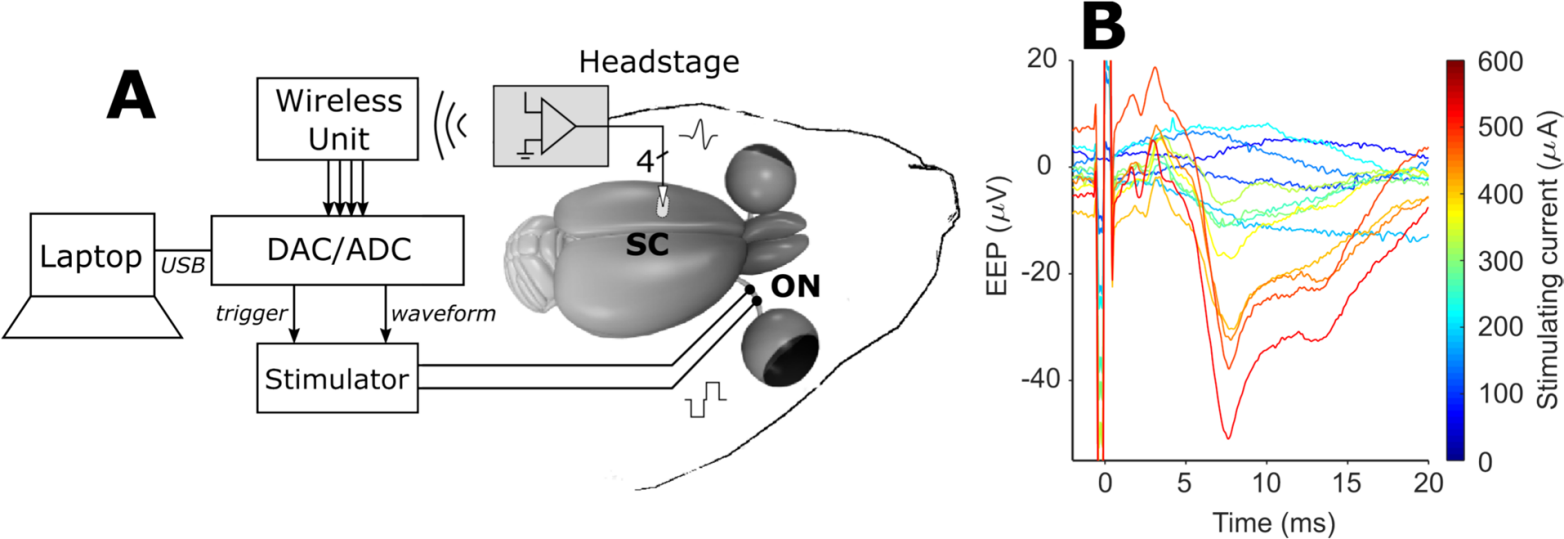
Figure obtained from a previous publication [35]. Briefly, (**A**) shows the in vivo experimental setup used for delivering constant-current biphasic stimulus to the optic nerve (ON) of an anaesthetized rat. Electrically evoked potentials (EEPs) were registered from the superior colliculus (SC) using a headstage amplifier connected wirelessly to a commercial digital-to-analog (DAC) converter. This DAC, controlled by a laptop computer via a USB port, was used to generate biphasic constant-voltage waveforms 500 µs in duration with inter-phase time set to 10 µs with amplitudes varying between 0 and 3.6 V. In this setup, the stimulator is the modified Howland voltage-to-current converter, with 1 mA·V.^−1^ transconductance, followed by the electrode shorting setup, both described in detail in the present paper. (**B**) illustrates ensemble averages of EEPs thus recorded with various stimulus amplitudes. The electrical artifact caused by the stimulus can be noticed at t = 0

Additionally, an in vivo test employing the Howland configuration described here was previously reported [35], which shows the validation of the chosen voltage-to-current converter and electrode shorting circuitry for pre-clinical research. In a previous publication, Barriga-Rivera and co-workers used a Micro 1401–3 data acquisition unit (Cambridge Electronic Designs Limited, Cambridge, UK) to generate biphasic constant-voltage waveforms with 500 µs phase and 10 µs phase delay times. The amplitude ranged from 0.0 to 3.6 V to generate current waveforms with amplitude comprised between 0 and 3.6 mA respectively. The Micro 1401–3 was used for this setup, instead of the presented FPGA design and signal conditioning circuity. The output of the Howland amplifier was interfaced to a pair of silver hook electrodes implanted into the optic nerve of Wistar rats. Electrically evoked potentials were successfully recorded from the superior colliculus, as shown in Fig. 11.

## Discussion

The use of electrical stimulation has unlocked treatments to many health problems including hearing and visual deficits among others. For example, the cochlear implant has recently reached a million recipients [52], and retinal implants keep aiming at mimicking the success of the bionic ear with research efforts from many viewpoints. Interestingly, the use of high-frequency electrostimulation is promising assistance with better selecting different neuron types which exhibit morphological and functional differences, such as the ON and OFF retinal ganglion cells [39]. However, these stimulation strategies rely on the use of trains of biphasic pulses, as most neurostimulators have been designed to deliver constant-current pulses. It is expected that stimuli with arbitrary waveforms are able to unlock more precise and selective recruitment of neurons, as these waveforms may produce targeted activation while reducing the amount of energy needed. But arbitrary waveform stimulation may have other applications such as the delivery of distant stimulation via interfering electrical currents [18]. In this case, by creating electric fields temporally patterned, it is possible to achieve neuromodulation at an intermediate location; arbitrary waveforms can enable more precise interfering patterns, thus facilitating applications such as transcranial brain stimulation. To aid research in these fields, the arbitrary neural stimulator reported in this work was built using an inexpensive open-source FPGA board and basic analog electronic circuits. We have presented an inexpensive instrument that can be easily built in any electronic laboratory.

This device was conceived for experimental research in retinal electrostimulation and included two isolated channels, as a low channel count is sufficient for addressing many research questions in the field. Nevertheless, the system can be easily scaled up to different channel counts by connecting several of them in a parallel manner and using the trigger signals defined for these purposes. Note that the target electrodes in retinal prostheses have diameters in the order of few hundreds of micrometers. Thus, design requirements were analyzed to target these types of electrodes, which present typical impedance values at 1 kHz from few kilohms to few tens of kilohms [37]. In other research scenarios where the electrodes are made from microwires, classically employed in behavioral neuroscience to measure local field potentials, the typical impedance may reach up to few hundreds of kilohms [38]. These electrodes are normally driven by substantially lower current levels (50–100 µA) to avoid permanent damage produced by exceeding the electrochemical safety limits. However, given the high impedance they may exhibit, the total voltage across the electrodes may be close to the compliance limits of the neurostimulator. If a wider compliance voltage is required (as in the previously discussed case [38]), the voltage supply of the transconductance amplifier used for the voltage-to-current conversion can slightly increase to up to ± 20 V; if this is not sufficient for a given application, the TL072ACP chip can be replaced by a high-voltage operational amplifier such as the ADA4700 (Analog Devices, Wilmington, USA), which can operate at up to ± 50 V. Under these conditions, a 500 k Ω impedance could be driven with an electric current up to 100 µA.

Certain components can introduce unpredictable DC levels that will propagate to the current waveforms. Coupling capacitors have the potential to reduce these DC levels at the cost of modifying the frequency response of the neurostimulator, resulting in unwanted reshaping of the stimuli signals. Furthermore, the coupling capacitors will act as high-pass filters with uncontrolled cutoff frequencies, as these cutoff frequencies would ultimately depend on the impedance of the electro-tissue interface. To address this limitation, we have adopted a software-focused strategy, in which we compensate for the detected offset during the generation of the signal samples. Likewise, charge balance requirements can be addressed by carefully designing the arbitrary waveforms to comply with the electrochemical windows of the electrode materials.

To provide good electrical isolation, a low-distortion wide-band isolation amplifier was used. As this amplifier is typically powered at ± 15 V, if larger voltage compliance levels are required between the electrode terminals, the output signal must be adapted by either including an additional amplification stage between the isolation amplifier and the transconductance amplifier or by increasing the gain of the Howland configuration. Note that a repertoire of alternatives exists to the AD215AY circuit; these include isolation amplifiers with larger noise levels. A relatively simple strategy to obtain improved signal-to-noise ratios in the electrical isolation stage relies on increasing the amplitude of the input signal while proportionally decreasing the transconductance of the voltage-to-current converter. Here, the use of DC-DC converters may be perceived as a relevant limitation, as these devices introduce high-frequency noise caused by the characteristic rapid internal switching. While isolated DC-DC converters provide electrical isolation beyond the 1000 VDC required in neural stimulation, the characteristic noise may transfer to the current signal via power supply of the electronic subsystems or via electromagnetic coupling. The first noise-transfer mechanism can be reduced by including capacitors able to compensate for these quick variations of the electric potential. The second can be ameliorated by placing DC-DC converters relatively far from neighboring PCB tracks. Alternatively, electrically isolated power supply can be provided using batteries, thus increasing the size and weight of the device while limiting the usability of the device to the charge of the battery.

The design of the two-channel arbitrary neurostimulator presented here can be tailored to different applications by slightly modifying some of the components used. In addition, multiple devices can be easily wired in parallel to deliver high channel count arbitrary electrical stimulation. The isolated end of the neurostimulator has been previously tested in vivo [35] showing excellent results. Further testing is required to demonstrate the efficacy of particular current waveform and stimulation strategies, for example, for the preferential activation of retinal ganglion cells [42] or for the non-invasive activation of brain structures [53].

Future work on this topic can proceed in different ways. First of all, although the development presented here is only intended for pre-clinical use, it has the potential to follow regulatory pathways for commercialization as a medical device; for that, as a general rule, the design and validation are recommended to be carried out following the ISO-13485 standard. For the FPGA, the gateware can be considered a software component; if so, IEC-62304 is to be followed. Consequently, the verification strategy proposed here is of relevant application: Since the testbench for the system is already structured according to industry-standard best practices, a predictor block that anticipates expected outputs could be added, so constrained random testing could be performed. This way, the required code coverage targets could be more easily reached, enabling compliance even with validation requirements for medical class III devices. Another avenue for future development would focus on increasing the capacity and capabilities of the neurostimulator. For example, using a similar command set, a massively parallel neurostimulator with a high number of channels could be developed, reusing many of the modules of the current stimulator. Finally, the presented neurostimulator, or parts of it, could be integrated into an Application Specific Integrated Circuit (ASIC), to pave the way for a future implantable device able to deliver arbitrary, case-specific, current waveforms.

## Supplementary Information

Below is the link to the electronic supplementary material.

Supplementary file1 (M 3 KB)

Supplementary file2 (PY 14 KB)
